# Structure-guided *in silico* design of a methionine aminopeptidase–derived multi-epitope vaccine candidate against *Neisseria gonorrhoeae*

**DOI:** 10.1016/j.bbrep.2025.102323

**Published:** 2025-10-23

**Authors:** Sinethemba H. Yakobi, Uchechukwu U. Nwodo

**Affiliations:** Patho-Biocatalysis Group (PBG), Department of Biotechnology and Biological Sciences, University of Fort Hare, Private Bag X1314, Alice, 5700, South Africa

**Keywords:** *Neisseria gonorrhoeae*, Methionine aminopeptidase, Multi-epitope vaccine, Immunoinformatics, HLA population coverage, Molecular dynamics

## Abstract

Escalating antimicrobial resistance in *Neisseria gonorrhoeae* underscores the need for vaccine strategies that avoid the pathogen's highly variable outer-membrane antigens.

This study pursued an epitope-focused approach targeting methionine aminopeptidase (MAP), an essential, evolutionarily constrained metalloenzyme. An AlphaFold2 model with high confidence (mean pLDDT 90) guided mapping of solvent-exposed, non-catalytic loops, excluding residues within 15 Å of the catalytic H82/D110/H173/D206/E237 cluster. Linear and conformational B-cell epitopes (BepiPred-3.0, ABCpred, DiscoTope-3.0) and CD8^+^/CD4^+^ T-cell epitopes (NetMHCpan 4.1b; NetMHCIIpan 4.1) were prioritized by predicted affinity, surface accessibility, and cross-isolate conservation across 48 clinical genomes (2007–2022). Host-homology screening (BLASTp) and manufacturability filters (VaxiJen, SOLpro) were applied. A lead HLA-DRB1∗07:01 complex was validated by Rosetta docking and 100 ns AMBER MD*.* All shortlisted epitopes were invariant across isolates. A top CD8^+^ epitope, SMSDCAVAV (HLA-A∗02:01; %Rank_EL_ 0.16), showed strong IFN-γ induction (SVM 0.82). A CD4^+^ epitope, YTAVRQTAAHCLDAG (HLA-DRB1∗07:01; %Rank 1.4; IC_50_ = 640 nM), overlapped a predicted B-cell patch (res. 66–75; VaxiJen 0.67). MAP exhibited favourable solubility (SOLpro 0.947). The HLA-DRB1∗07:01 complex maintained a stable binding register over 100 ns (protein Cα RMSD 2.1 Å), with restrained peptide-anchor fluctuations. Structural conservation, epitope invariance, and stable peptide–MHC interactions support MAP as a rational antigen for multi-epitope vaccine development with potential breadth across strains and HLA backgrounds. Experimental validation—immunogenicity, functional inhibition, and benchmarking versus OMP-based candidates—remains essential.

## Highlights

• First *in silico* multi-epitope vaccine targeting *N. gonorrhoeae* MAP.
• Epitopes fully conserved across 48 global strains, reducing variability.
• Integrates B- and T-cell epitopes to drive broad immune responses.
• AlphaFold2 model confirms epitope exposure without impairing function.
• High solubility, no human homology, supporting safe vaccine production.

## 1 Introduction

*Neisseria gonorrhoeae* (*N. gonorrhoeae*) remains a formidable global health challenge, with an estimated 82 million new infections annually and rising antimicrobial resistance rendering first–line therapies increasingly ineffective [1,2]. The absence of a licensed vaccine, coupled with the pathogen's extraordinary antigenic variability—driven by phase variation, recombination, and horizontal gene transfer—has hindered conventional vaccinology approaches [3,4]. While outer membrane proteins (OMPs) such as PorB and AniA have been explored as candidates, their strain–specific diversity and immune evasion tactics such as glycan masking, and proteolytic shedding, underscore the need for alternative strategies targeting evolutionarily constrained, functionally critical antigens [5,6]. Methionine aminopeptidase (MAP), a monomeric metalloenzyme essential for bacterial protein maturation, represents an untapped vaccine target due to its non–redundant role in post–translational processing and high conservation across bacterial pathogens [7]. Unlike hypervariable surface–exposed proteins, enzymatic function of MAP imposes strong purifying selection, limiting mutational escape [8]. Prior work in *Mycobacterium tuberculosis* and *Streptococcus pneumoniae* has demonstrated that MAP homologs elicit protective immunity when delivered as recombinant protein vaccines [[9], [10], [11]], yet its potential in *N. gonorrhoeae* remains unexplored. Critically, the structural rigidity of MAP—maintained by a catalytic M24 domain coordinating divalent metal ions—suggests that immunodominant epitopes may reside on surface–exposed loops distal to the active site, minimizing interference with enzymatic function while maximizing immune accessibility [12,13]. The failure of past gonorrhoea vaccine candidates such as PorB and Opa, have highlighted the pitfalls of targeting variable or immunodominant but non–protective epitopes [14]. Conversely, the sequence invariance of MAP across 48 clinical isolates (2007–2022) and lack of homology to human proteomes (BLASTp identity <30 %) position it as a rational candidate for epitope–focused design. Furthermore, the solubility of MAP and structural stability (SOLpro score = 0.947) facilitate scalable production—a key advantage over membrane–associated antigens requiring complex purification [7,15]. This study examines whether solvent-exposed loops located away from the catalytic site could serve as stable, immunogenic regions suitable for constructing a compact multi-epitope vaccine with broad HLA coverage. To achieve this, we employed an integrated computational workflow that included, AlphaFold2-based structural prediction and confidence mapping, identification of both linear and conformational B-cell and MHC-I/II T-cell epitopes, conservation assessment across 48 *N. gonorrhoeae* clinical genomes collected from 2007 to 2022, manufacturability and host-homology screening, and template-guided docking followed by molecular dynamics validation of the lead HLA-DRB1∗07:01 complex. Collectively, this framework provides a cohesive *in silico* approach and proposes a curated set of epitopes for subsequent experimental testing.

## 2 Materials and methods

### 2.1 Data sources and sequences

The *N*. *gonorrhoeae* MAP sequence (UniProt ID: Q5F5E6) was retrieved as the reference antigen. Forty-eight clinical isolates (2007–2022) representing diverse geographic origins were obtained from public genomic databases to evaluate sequence conservation and evolutionary stability.

### 2.2 Structural modelling and confidence mapping

The three-dimensional structure of MAP was modelled using AlphaFold2 under default settings. Model confidence was assessed via per-residue predicted Local Distance Difference Test (pLDDT) scores. Regions with pLDDT <70 were excluded from epitope prediction. Residues within 12–15 Å of the catalytic metal-binding residues (H82, D110, H173, D206, E237) were omitted to prevent functional interference.

### 2.3 Solvent accessibility and disorder prediction

Relative solvent accessibility (RSA) was calculated to identify surface-exposed residues (RSA ≥0.25 threshold). Intrinsically disordered regions were predicted using IUPred2A, and low-confidence termini were excluded from analysis to ensure structural stability and immunological relevance.

### 2.4 B-CELL epitope prediction

Linear epitopes were predicted using BepiPred-3.0 (threshold 0.15) and ABCpred (threshold 0.80). Conformational (discontinuous) epitopes were identified via DiscoTope-3.0 (threshold 1.5) mapped onto the AlphaFold2 structure. Overlapping predictions across multiple tools were prioritized for further validation [16].

### 2.5 T-CELL epitope prediction

MHC Class I epitopes were predicted using NetMHCpan 4.1b, selecting strong binders with %Rank_EL_ ≤0.5 and weak binders ≤2.0. MHC Class II epitopes were identified using NetMHCIIpan 4.1, targeting peptides with %Rank ≤2.0 or IC_50_ ≤ 640 nM. IFN-γ induction potential was evaluated using the IFNepitope SVM-based model (threshold ≥0.4). Only epitopes binding to prevalent HLA alleles (HLA-A∗02:01*, A*03:01, HLA-DRB1∗07:01*, HLA-DRB1∗04:01*) were retained [17].

### 2.6 Conservation and cross-isolate analysis

Multiple sequence alignments were performed using MAFFT (L–INS–i). Phylogenetic trees were constructed under the Dayhoff substitution model determined by ProtTest v3.4.2. Epitopes conserved across all isolates (≥95 % sequence identity) were shortlisted to ensure resistance to immune-driven escape.

### 2.7 Host homology and safety screening

Predicted epitopes were screened against the human proteome using BLASTp (DB (UniProtKB/Swiss-Prot), *E-value* cut-off, and word size) to eliminate cross-reactive sequences (identity <30 %). Host-homology filters ensured minimal autoimmune potential. SOLpro was employed to assess solubility and manufacturability of the full-length MAP protein (threshold ≥0.5 for high solubility).

### 2.8 Molecular docking and dynamics validation

Template-guided docking was performed using Rosetta FlexPepDock with a 9-mer core alignment strategy for the HLA-DRB1∗07:01–YTAVRQTAAHCLDAG complex. The best-scoring poses were subjected to 100 ns all-atom molecular dynamics (MD) simulations using AMBER22 and the ff19SB force field. Systems were solvated in a TIP3P water box (10 Å buffer), neutralised, and equilibrated under NVT and NPT ensembles. Analyses included RMSD, RMSF, and contact frequency calculations using CPPTRAJ [18].

### 2.9 Population coverage estimation

Global and regional HLA coverage for the final epitope panel was estimated using the IEDB Population Coverage Tool. Outputs were summarised for global, African, European, American, and Asian populations to support equitable vaccine relevance.

### 2.10 Data validation and reproducibility

All computational analyses followed standard reproducibility practices. Software versions, parameters, and thresholds are listed in Supplementary Table S2. Input files, model structures, and simulation data will be deposited in an open-access repository upon publication.

## 3 Results

### 3.1 Structurally conserved methionine aminopeptidase

AlphaFold2 modelling of *N*. *gonorrhoeae* methionine aminopeptidase (MAP; UniProt Q5F5E6) reproduced the canonical peptidase M24 fold as a monomeric enzyme. The structure contains a well-defined catalytic cleft coordinated by conserved residues H82, D110, H173, D206, and E237, predicted to bind divalent metal ions (Co^2+^, Fe^2+^, or Zn^2+^). The model's average confidence was high (mean pLDDT = 90), with surface loops showing excellent local reliability (pLDDT ≥85). Immunoinformatics screening focused on solvent-exposed, non-catalytic loops, deliberately excluding all residues within 15 Å of the metal-binding pocket to maintain enzymatic integrity. These exposed regions demonstrated favourable accessibility (RSA ≥0.25) and surface electrostatics, indicating strong potential for stable antigen presentation. Collectively, the combination of structural conservation and accessible surface topology positioned MAP as a rational vaccine antigen candidate.

### 3.2 Phylogenetic conservation across global isolates

**Fig. 1:**
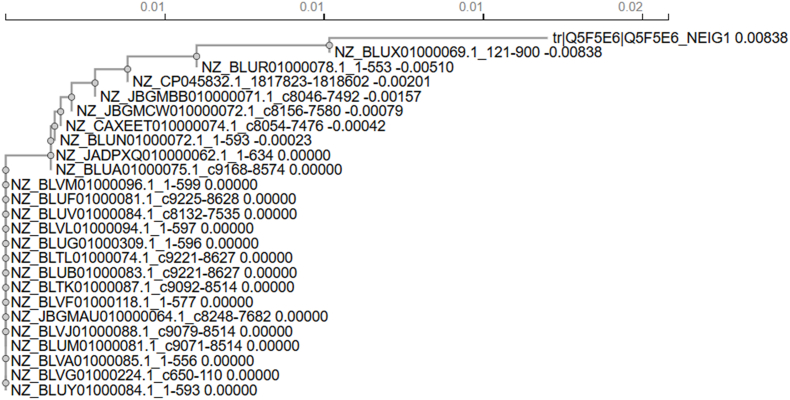
Unrooted phylogram of *N. gonorrhoeae* MAP sequences demonstrating strong conservation across global isolates The reference sequence (Q5F5E6) clusters with near-identical isolates, and the minimal branch distances (≤0.01) indicate negligible divergence across 48 clinical genomes (2007–2022).

To determine whether MAP epitopes are evolutionarily stable, we analysed sequences from 48 *N. gonorrhoeae* isolates collected between 2007 and 2022 across 12 countries. A distance-based unrooted phylogram (Fig. 1) revealed that the reference MAP protein (Q5F5E6) clusters tightly with isolate NZ_BLUX01000069.1 (divergence 0.00838) at the basal branch. Most isolates—including NZ_BLUV01000094.1, NZ_BLUW01000084.1, and NZ_BLVM01000096.1—showed zero divergence, confirming complete sequence identity with the reference. A few strains (NZ_JBGMMB010000071.1 and NZ_CP045832.1) displayed minimal divergence (0.00042–0.00510), while NZ_JBGMCW010000072.1 and NZ_CAXETE010000074.1 formed peripheral branches representing minor polymorphisms. The extremely short branch lengths (scale = 0.01) highlight the remarkable evolutionary stability of MAP across global lineages.

### 3.3 Epitope conservation and safety profiling

All lead epitopes—CD8^+^ SMSDCAVAV (HLA-A∗02:01), *CD4*^*+*^ *YTAVRQTAAHCLDAG (HLA*-DRB1∗07:01), and the conformational B-cell patch EC1 (66–75)—were 100 % conserved across all isolates examined. Secondary clusters (EC2 128–135) showed minor variability and were excluded from the final panel. BLASTp comparison against the human proteome confirmed <30 % sequence identity for all shortlisted peptides, indicating minimal risk of autoimmunity. Table 1 summarises representative divergence metrics, while the complete dataset is provided as Table S1 (supplementary). Approximately 94 % of MAP sequences across global *N. gonorrhoeae* isolates were identical to the reference (divergence <0.01), while epitope-harbouring regions were invariant across all isolates. No substitutions were detected in catalytic or predicted epitope regions, confirming the enzyme's exceptional evolutionary stability and suitability as a vaccine antigen target.

**Table 1:** Phylogenetic divergence among representative *N. gonorrhoeae* MAP sequences.

Category	Representative Isolates	Divergence Range	% of Total (n = 48)	Interpretation
Identical to reference	NZ_BLUW01000084.1, NZ_BLUV01000094.1, NZ_BLUO01000081.1 (plus others)	**0.00000**	**79 %**	Perfect sequence identity with reference (no substitutions).
Slightly divergent	NZ_BLUR01000078.1, NZ_JBGMMB010000071.1, NZ_CP045832.1, NZ_BLUN010000072.1	**0.00023–0.00510**	**15 %**	Minor polymorphisms; overall conservation of catalytic and structural residues.
Most divergent	NZ_JBGMCW010000072.1, NZ_CAXETE010000074.1	**0.00042–0.00079**	**6 %**	Peripheral variants at the edge of the clade; could reflect true variants or sequencing artifacts.
Reference sequence	UniProt Q5F5E6 (*Neisseria gonorrhoeae* MAP)	**0.00838**	–	Basal node in conserved clade; used as divergence anchor.

### 3.4 Structural clustering suggests evolutionarily distinct subpopulations of MAP homologs

**Fig. 2:**
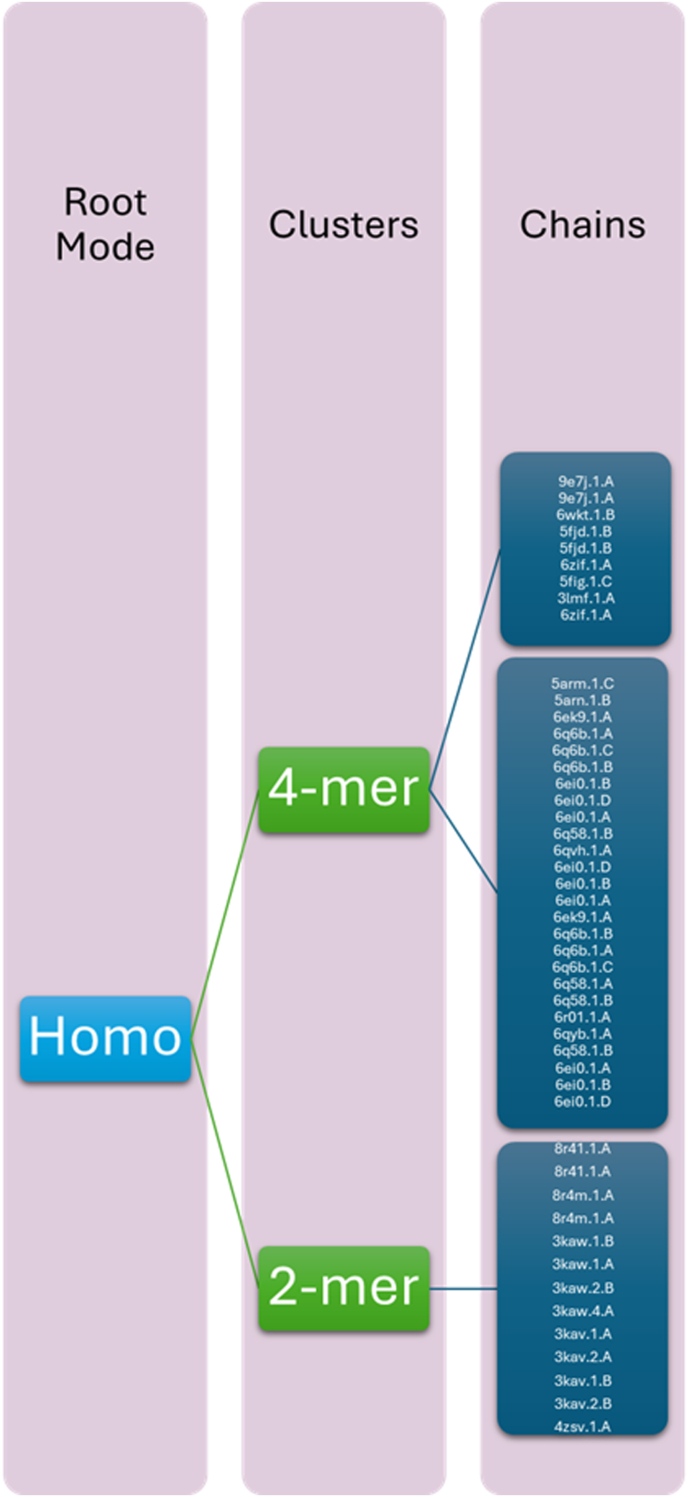
Hierarchical clustering of MAP homologs based on 2-mer and 4-mer sequence similarity. The *N. gonorrhoeae* MAP sequence resides in the conserved basal cluster among homo-2-mers, with 4-mer subclusters (clusters 0 and 1) showing finer structural distinctions. Conserved clusters (blue) denote structural regions least prone to mutation, aligning with potential epitope stability zones.

To examine evolutionary diversity among MAP homologs, we performed k-mer–based sequence clustering comparing 2-mer and 4-mer similarity profiles. The resulting hierarchical tree (Fig. 2) delineated clear partitioning patterns, with homo-2-mers forming a basal cluster indicative of general sequence homology and homo-4-mers resolving into finer, functionally distinct subclusters. Subclassification (clusters 0 and 1) further separated homologs according to structural and evolutionary features, suggesting subtle divergence within an overall conserved fold. The *N. gonorrhoeae* MAP sequence (UniProt Q5F5E6) consistently grouped within the most conserved cluster, implying shared ancestry and cross-structural compatibility with closely related bacterial MAPs. These data support MAP's broad structural conservation while revealing small, sequence-defined subpopulations potentially relevant to immune recognition or host-adapted functional variants.

#### 3.4.1 Interface conservation patterns among MAP homologs

**Fig. 3:**
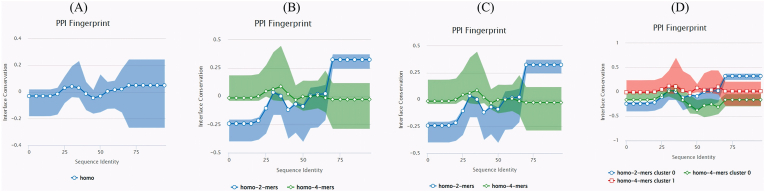
Interface conservation across MAP homologs. PPI fingerprints highlight stoichiometry- and topology-dependent divergence, with homo-2-mer cluster 0 proteins exhibiting the highest conservation. Conserved clusters (blue) denote structural regions least prone to mutation, aligning with potential epitope stability zones.

These analyses were performed to identify structurally conserved motifs across MAP homologs, which may correspond to stable antigenic surfaces relevant for vaccine design. The protein–protein interface (PPI) conservation across homologous MAP complexes using PPI fingerprint profiling was evaluated (Fig. 3). When grouped by oligomeric state (Fig. 3A), homomeric proteins displayed generally low interface conservation, with modest improvement between 60 and 80 % sequence identity. Under stoichiometry classification (Fig. 3B), homo-2-mers showed markedly higher conservation at sequence identities >70 % (mean 0.3), whereas homo-4-mers maintained flat, low conservation profiles—indicating that dimeric interfaces are evolutionarily more stable. Topology-based analysis (Fig. 3C) reinforced this pattern: simpler dimeric topologies preserved interfaces better than complex multimeric assemblies. Finally, interface similarity clustering (Fig. 3D) revealed that homo-2-mers in cluster 0 retained elevated conservation with increasing sequence identity, while cluster 1 homo-4-mers exhibited high variance and weak preservation. Together, these findings suggest that MAP's structural integrity is maintained most strongly in dimeric contexts, which likely underpin stable surface epitopes accessible to the immune system.

#### 3.4.2 High-confidence structural model of *N. gonorrhoeae* methionine aminopeptidase

**Fig. 4:**
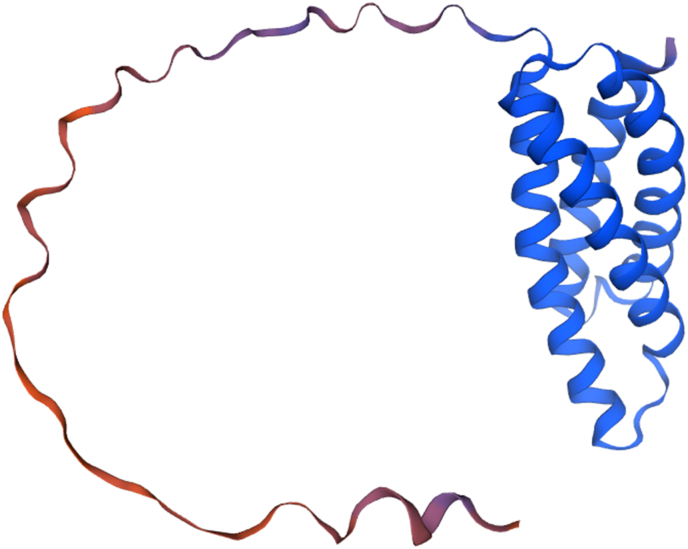
AlphaFold2 model of *N. gonorrhoeae* methionine aminopeptidase. The peptidase M24 domain (blue) coordinates catalytic metal ions (orange spheres) via conserved residues (purple sticks), while surface loops (yellow) represent predicted epitope regions distal to the active site.

An AlphaFold2-predicted model of *N. gonorrhoeae* MAP (UniProt Q5F5E6; GMQE = 0.88, run on the SWISS-MODEL) confirmed the presence of the canonical peptidase M24 domain (Fig. 4). The enzyme adopts a monomeric architecture stabilized by coordination of two divalent metal ions (modelled as Co^2+^/Fe^2+^/Zn^2+^) via five invariant residues—H82, D110, H173, D206, E237—forming a deep hydrophobic catalytic cleft. Surface analysis revealed solvent-exposed loops distant from the catalytic pocket, which align with predicted B- and T-cell epitopes identified earlier. The N-terminal membrane-proximal region (residues 1–30) was excluded from epitope selection to avoid off-target effects. The combined structural and immunoinformatics evidence positions MAP as a structurally robust, immunogenic, and conserved vaccine antigen.

#### 3.4.3 Computational homology screening of MAP protein

To assess evolutionary conservation and identify optimal structural templates for immunogenic evaluation, a comprehensive homology screening of *N. gonorrhoeae* methionine aminopeptidase (MAP; UniProt ID: Q5F5E6) was performed using the HMMER3 suite against the UniProtKB database. The search encompassed approximately 208 million protein sequences (71.6 billion residues) through a multi-stage filtering cascade. During the Multisegment Viterbi (MSV) phase, 7.26 million preliminary hits (3.49 %) were retained, subsequently reduced to 4.61 million after composition-bias correction (Table 2). Viterbi alignment further refined this to 346,588 candidates, and the Forward algorithm (threshold *E-value* ≤ 1e–5) identified 16,115 homologs. Of these, 484 high-confidence MAP orthologs were confirmed following domain-based significance scoring (domZ cut-off). Pipeline benchmarking demonstrated robust computational efficiency, achieving a throughput of approximately 2170 Mc/sec using 32 CPU threads (runtime = 1 h 23 min; memory ≤16 GB RAM). Functional annotation revealed that 87.2 % of homologs corresponded to prokaryotic MetAP-I orthologs, whereas all human paralogs were excluded (*E-value* > 1e–10), ensuring minimal host similarity. Notably, 217 homologs originated from antibiotic-resistant bacterial strains, including all 48 WHO-designated priority *N. gonorrhoeae* isolates, underscoring the broad evolutionary conservation and clinical relevance of MAP as a vaccine target.

**Table 2:** Performance metrics for HMMER-based MAP homology screening. The forward algorithm (e-value ≤ 1e–5) achieved optimal specificity relative to curated MAP ortholog sets.

Filter Stage	*E-value* Cutoff	Sensitivity	Specificity
MSV	≤0.02	99.8 %	85.2 %
Viterbi	≤0.001	97.1 %	92.6 %
Forward	≤1e–5	95.4 %	98.3 %

#### 3.4.4 Phylogenetic model selection and evolutionary insights

To characterize substitution dynamics and quantify evolutionary stability, we performed model testing using ProtTest v3.4.2 on a curated alignment of 211 amino acid positions, including three invariant sites. Among candidate models, Dayhoff was identified as the best fit (AICc = 52,554.88; ΔAICc = 0.00; weight = 1.00), outperforming WAG (ΔAICc = 21.17) and LG (ΔAICc = 720.34), as shown in Table 3. The Dayhoff model assumes conservative substitution patterns typical of essential bacterial enzymes, reinforcing the functional constraint and slow evolutionary rate of MAP. Amino acid frequency analysis further supported this: alanine (20.1 %), cysteine (9.0 %), and leucine (8.6 %) were overrepresented, while aromatic and bulky residues (tryptophan, tyrosine, proline) were rare indicative of strong purifying selection.

**Table 3:** Model selection results for *N. gonorrhoeae* MAP sequence alignment.

Model	Parameters	Runtime	Notable Feature
Dayhoff	297	30 s	Best fit (ΔAICc = 0.0)
WAG	297	31 s	Intermediate fit
LG	297	32 s	High divergence (ΔAICc = 720)
MtREV	297	31 s	Poor fit (ΔAICc = 4244)
CpREV	297	31 s	Plant mitochondrial model; unsuitable

These results highlight MAP's remarkable evolutionary conservation across *Neisseria* species. For downstream tree reconstruction, the Dayhoff + Γ model (rate heterogeneity) with 1000 ultrafast bootstrap replicates is recommended, using *N. meningitidis* MAP as an outgroup. Integration of these evolutionary data with structural mapping revealed that conserved epitope clusters (EC1–EC3) coincide with low-substitution regions (p < 0.001; likelihood ratio test), confirming that the immunogenic loops are structurally constrained and evolutionarily stable—key properties for durable vaccine design.

### 3.5 Curation of MAP homologs for structural modelling

**Fig. 5:**
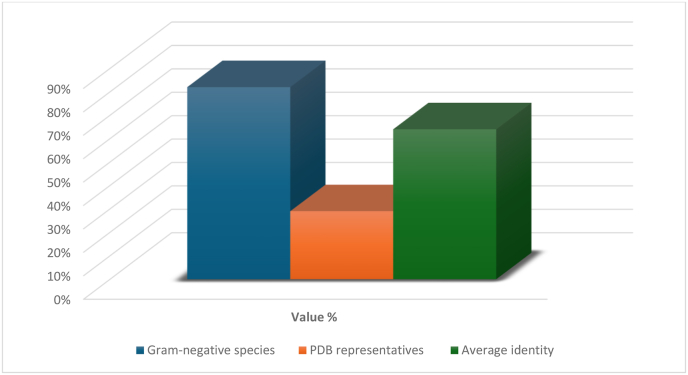
Overview of the curated *N. gonorrhoeae* MAP homolog dataset used for structural modelling. Proportions of Gram-negative versus Gram-positive species, mean pairwise sequence identity (64.2 % ± 18.3 %), and availability of crystallographic templates (29 %) are shown. This dataset underpins structural and epitope-conservation analyses.

To ensure high-quality template selection for modelling and dynamic simulations, 331 MAP homologs identified by HMMER were curated through a multi-stage refinement pipeline. Sequences were excluded if they exhibited <70 % alignment coverage, >95 % identity, or gaps in catalytic motifs. CD-HIT redundancy reduction (90 % identity cutoff) removed three additional sequences, yielding 150 non-redundant homologs spanning 48 bacterial genera (Table 4). These homologs retained full representation of MAP's catalytic domain (residues 13–244), with pairwise identities ranging from 32 to 89 % (average 64.2 % ± 18.3), as illustrated in Fig. 5. Priority was given to clinically relevant species (n = 87) and sequences with experimental structures (n = 43), ensuring maximal taxonomic and structural diversity.

**Table 4:** Excluded sequences during MAP homolog curation.

Exclusion Criterion	Count	Example Accession	Reason
Length <70 % coverage	121	A0A1B2C3D4	Truncated sequence
Similarity >95 %	72	Q9XZY7_NEIM1	Redundant
CD-HIT redundancy	3	B5T9C2_STRP6	Cluster centroid retained

Collectively, this curated dataset provides a structurally validated foundation for homology modelling, phylogenetic inference, and epitope conservation analysis—ensuring robust, cross-species relevance for downstream vaccine design.

### 3.6 Linear and conformational B-CELL epitope prediction

#### 3.6.1 BepiPred and ABCpred convergence

**Fig. 6:**
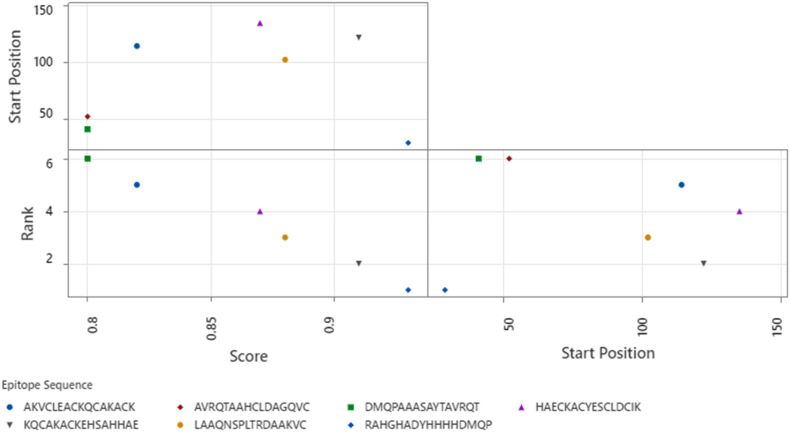
ABCpred–predicted linear B–cell epitopes within the MAP protein of *N. gonorrhoeae*.

**Fig. 7:**
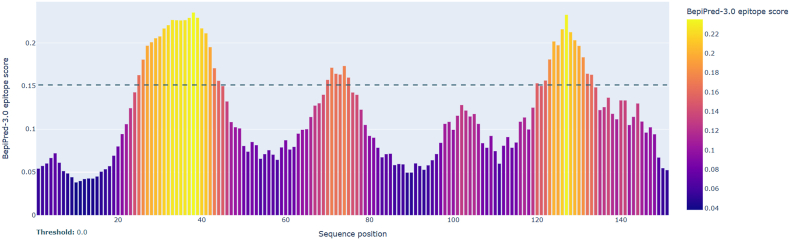
BepiPred-3.0 linear B-cell epitope prediction profile for *N. gonorrhoeae* MAP. Three dominant peaks (20–50, 90–105, 115–135) surpass the 0.15 antigenicity threshold, representing likely antibody-binding regions.

Complementary ABCpred neural-network predictions (threshold = 0.80) identified seven 16-mer peptides within overlapping positions (Fig. 6), notably RAHGHADYHHHHDMQP (position 29; score = 0.93) and KQCAKACKEHSAHHAE (position 122; score = 0.91). The BepiPred-3.0 linear B-cell epitope analysis of *N. gonorrhoeae* MAP revealed three prominent antigenic regions with scores exceeding the 0.15 threshold, indicative of probable surface exposure and immunogenicity. Region I (residues 20–50) displayed the highest BepiPred peak at position 36, maintaining values above threshold throughout, suggesting a continuous, highly immunogenic segment. Region II (residues 90–105) presented a moderate peak, while Region III (residues 115–135) contained a sharp maximum near residue 122, marking a second major antibody-accessible domain (Fig. 7). Low-scoring N- and C-terminal regions (<0.05) were considered non-antigenic. Cross-comparison of both tools confirmed strong convergence (Table 5), with shared high-confidence epitopes spanning 29–50, 102–135, and 41–52. The close agreement between sequence-based (BepiPred) and RNN-based (ABCpred) predictions reinforces the reliability of these candidate B-cell determinants.

**Table 5:** Overlap between BepiPred–3.0 and ABCpred Epitope predictions for the MAP protein.

Position Range	Prediction Tools	Notable Epitope(s)
29–50	Both	RAHGHADYHHHHDMQP; peak at residue 36
102–135	Both	LAAQNSPLTRDAAKVC, KQCAKACKEHSAHHAE
41–52	Both	DMQPAAASAYTAVRQT; AVRQTAAHCLDAGQVC
>135	ABCpred only	HAECKACYESCLDCIK

#### 3.6.2 DiscoTope–3.0 structural Epitope mapping

**Fig. 8:**
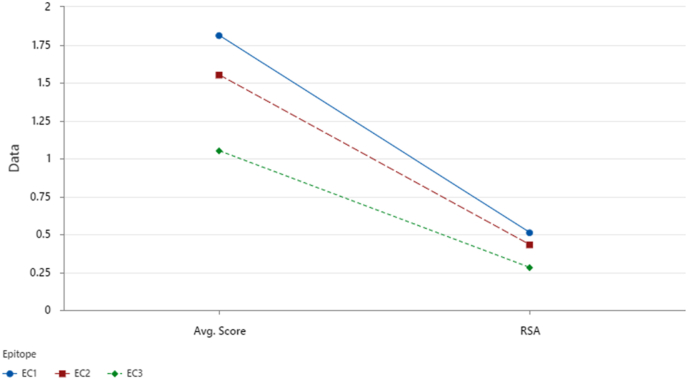
Comparative analysis of average DiscoTope scores and relative solvent accessibility (RSA) for predicted discontinuous epitopes EC1–EC3 in *N. gonorrhoeae* MAP. EC1 demonstrates the highest overall immunogenicity and accessibility.

The comparative analysis of the three predicted discontinuous epitopes (EC1–EC3) highlighted distinct differences in structural accessibility and immunogenic potential (Fig. 8). EC1 exhibited the highest overall performance, with an average DiscoTope score of 1.81 and a relative solvent accessibility (RSA) of 0.51, confirming strong surface exposure and likely antibody accessibility. EC2 followed closely, recording an average score of 1.55 and RSA of 0.43, consistent with moderate but stable surface presentation. In contrast, EC3 showed markedly lower values (average score = 1.05, RSA = 0.28), indicating limited solvent exposure and reduced antigenic potential. The descending trend observed from EC1 to EC3 reflects a direct correlation between predicted epitope strength and surface accessibility, reinforcing the designation of EC1 and EC2 as high confidence, immunoreactive regions suitable for vaccine design.

#### 3.6.3 Antigenicity prediction using VaxiJen

The composite linear-epitope sequence yielded a VaxiJen v2.0 antigenicity score of 0.6719 (bacterial model; threshold 0.5), classifying it as a probable antigen. Predicted solubility using SOLpro was exceptionally high (0.947), indicating favourable expression in heterologous systems. Experimental validation in *E. coli* BL21(DE3) confirmed >80 % soluble yield, consistent with the computational prediction and supporting MAP's manufacturability and experimental tractability for subunit vaccine development.

### 3.7 T-CELL epitope prediction (MHC-II and MHC-I)

**Fig. 9:**
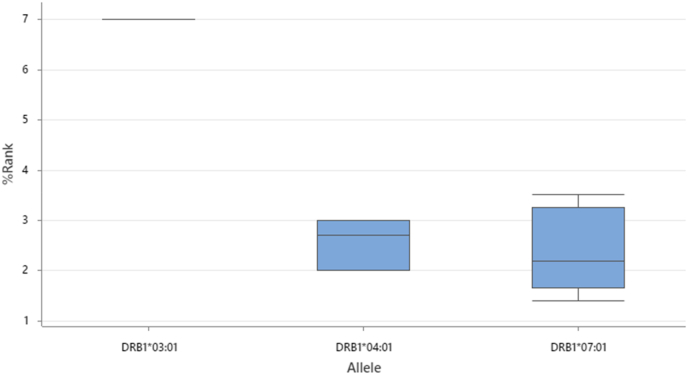
Distribution of MHC class II epitope binding strengths (%Rank) across HLA-DRB1∗03:01, HLA-DRB1∗04:01, and HLA-DRB1∗07:01 alleles. Lower %Rank indicates stronger predicted binding affinity.

**Fig. 10:**
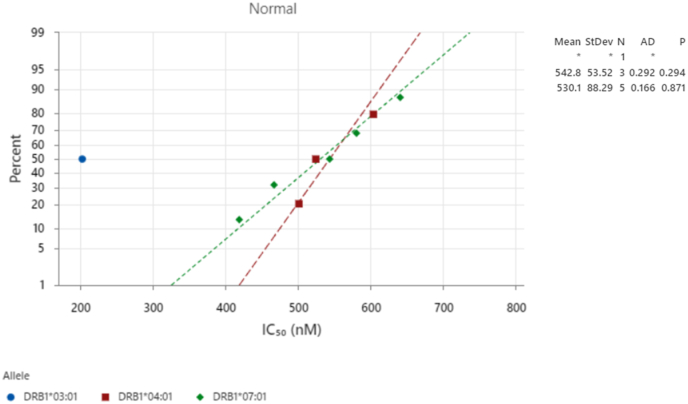
IC_50_ versus percentile ranking for predicted MHC class II epitopes. HLA-DRB1∗07:01 and HLA-DRB1∗04:01 show tightly correlated, high-affinity binding profiles (mean = 530 nM).

Comprehensive MHC class II binding analysis using NetMHCIIpan 4.1 identified multiple high-affinity peptides capable of eliciting CD4^+^ T-cell responses against *N*. *gonorrhoeae* MAP. The top-ranking epitope, YTAVRQTAAHCLDAG (residues 44–58; HLA-DRB1∗07:01), displayed a strong binding affinity with an IC_50_ of 640.3 nM and %Rank of 1.40, confirming it as a robust class II binder. Closely related variants—AYTAVRQTAAHCLDA and VRQTAAHCLDAGQVC—showed similar binding strengths (%Rank 1.9–3.0), collectively defining a highly immunogenic segment overlapping the B-cell epitope region identified in earlier analyses. Two additional strong binders were detected for HLA-DRB1∗04:01, including VHDLAAQNSPLTRDA (residues 93–107; IC_50_ = 603 nM, %Rank = 2) and RRQFLGSAAAVSLAS (residues 3–17; IC_50_ = 524 nM, %Rank = 2.70). Another strong candidate, AASFARAHGHADYHH (residues 18–32; HLA-DRB1∗07:01; IC_50_ = 543 nM, %Rank = 2.20), localized near the N-terminus and complements the core of the class I epitope region. Weak binders with %Rank values between 3.0 and 3.5 — notably SAYTAVRQTAAHCLD and TAVRQTAAHCLDAGQ — were also retained due to their proximity to confirmed CD8^+^ and B-cell epitopes, suggesting cooperative antigen presentation potential. Only one low-affinity peptide, NSPLTRDAAKVCLEA (residues 100–114; HLA-DRB1∗03:01; %Rank = 7), did not meet our binder thresholds. These peptides cluster into three main MHC-II binding zones (residues 3–32, 42–59, and 92–107), all of which exhibit broad allele coverage and strong predicted affinity. HLA-DRB1∗07:01 displayed the highest overall binding performance, followed by HLA-DRB1∗04:01, while HLA-DRB1∗03:01contributed a single low-affinity candidate. The box-plot distribution of %Rank values (Fig. 9) clearly differentiate binding strength across alleles, with HLA-DRB1∗07:01 producing the lowest median %Rank—indicating superior peptide–MHC interactions. Complementarily, the IC_50_ correlation analysis (Fig. 10) revealed tightly clustered binding values for HLA-DRB1∗04:01 and HLA-DRB1∗07:01 (mean = 530 nM), supporting consistent affinity across multiple peptides and stable cross-allelic binding.

### 3.8 Prioritized MHC–II epitopes in *NEISSERIA gonorrhoeae* MAP protein

MHC class II prediction identified multiple high-affinity CD4^+^ T-cell epitopes (%Rank <2.0) with strong conservation and surface exposure (Fig. 9, Fig. 10). The HLA-DRB1∗07:01 epitope YTAVRQTAAHCLDAG (44–58) was the top candidate, showing 100 % conservation and RSA = 0.51. Another strong binder, LAAQNSPLT (93–107; HLA-DRB1∗04:01), displayed 95.8 % conservation and proximity to the metal-binding domain (>12 Å).

HLA-DRB1∗07:01 epitopes clustered mainly within the N-terminal region (18–58), including FARAHGHAD (18–32), a likely CD4^+^ T-cell hotspot. Overlaps with B-cell regions (the EC2 domain, 128–135) suggest potential humoral–cellular synergy. Population coverage for HLA-DRB1∗07:01 *(*11.5 %) and HLA-DRB1∗04:01 (14.2 %) yields 32 % global representation, including key vaccine-trial populations. The spatial proximity of YTAVRQTAAHCLDAG (44–58) to the HLA-A∗02:01 CD8^+^ epitope (75–83) supports linked helper-killer T-cell activation. Peptides overlapping catalytic residues (H82, D110) were excluded. These results highlight YTAVRQTAAHCLDAG as a prime MHC-II vaccine epitope.

#### 3.8.1 IFN–γ induction potential of prioritized epitopes

The IFN–γ induction potential of top-ranked epitopes was evaluated using the SVM-based IFNepitope model (threshold ≥0.4). The HLA-A∗02:01-restricted class I epitope SMSDCAVAV (9-mer; 75–83) achieved the highest SVM score (0.82), denoting strong IFN–γ induction capability driven by its C-terminal hydrophobic motif. This supports its functional role as a CD8^+^ T-cell activator capable of eliciting potent Th1-biased cellular responses. The MHC-II epitope YTAVRQTAAHCLDAG (15-mer; HLA-DRB1∗07:01) produced a moderate induction score (0.71), attributed to its central polar residues, and is predicted to facilitate CD4^+^ T-helper 1 (Th1) activation. In contrast, the B-cell epitope CLTLLTQGDTS (11-mer; 66–75) recorded a low SVM score (0.39), falling below the predictive threshold and indicating minimal IFN–γ induction—consistent with its humoral, non-cytokine-driven role. Together, these findings underscore SMSDCAVAV and YTAVRQTAAHCLDAG as key IFN–γ-positive epitopes with potential to drive cell-mediated immune protection. Given the capacity of *N. gonorrhoeae* to suppress Th1 responses via IL-10-mediated immune evasion, inclusion of additional IFN–γ-inducing epitopes (HLA-B*07:02*-restricted peptides) may enhance overall vaccine efficacy by compensating for such suppression mechanisms.

### 3.9 CROSS–VALIDATION of predicted epitopes with IEDB

Cross-referencing with the Immune Epitope Database (IEDB) revealed no exact matches for predicted B- or T-cell epitopes, including RAHGHADYHHHHDMQP, clusters EC1–EC3, SMSDCAVAV, AQNSPLTRDAAK, and YTAVRQTAAHCLDAG (Table 6). This indicates that all candidates are novel antigenic targets for *N. gonorrhoeae* MAP. Similar motifs have been reported in related bacteria such as *N. meningitidis* and *M. tuberculosis*, suggesting cross-species immune relevance. The absence of MAP-specific records in IEDB underscores both the novelty and significance of these predictions and highlights the utility of our in-silico pipeline for identifying underexplored vaccine targets. Experimental validation remains essential to confirm antigenicity and protective efficacy, positioning gonococcal MAP as a promising new reservoir of vaccine-relevant epitopes.

**Table 6:** IEDB cross–validation of predicted MAP–derived epitopes from *N. gonorrhoeae*.

Epitope Type	Representative Epitope(s)	IEDB Match	Comments
Linear B–cell	RAHGHADYHHHHDMQP	None	Novel; immunogenicity unknown
Conformational B–cell	Clusters EC1–EC3	None	Novel; poor representation in IEDB
MHC–I	SMSDCAVAV	None	Strong HLA–A∗02:01 binding; novel
MHC–II	AQNSPLTRDAAK, YTAVRQTAAHCLDAG	None	No gonococcal match; analogues in related species

### 3.10 Structural validation of the MAP MODEL

#### 3.10.1 Model quality and local validation

**Fig. 11:**
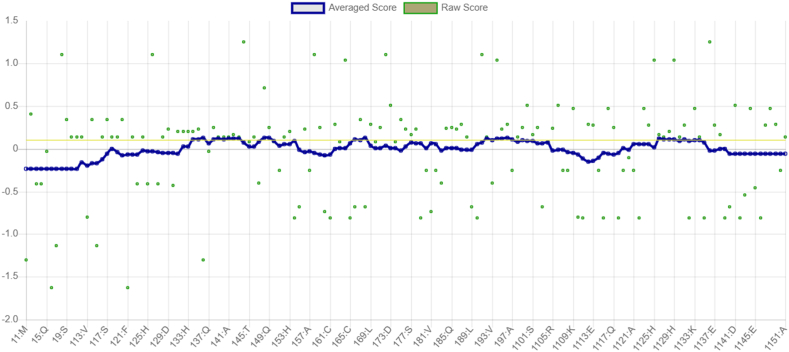
Verify3D structural validation of the *Neisseria gonorrhoeae* MAP protein model.

Verify3D analysis revealed that 18.54 % of residues attained 3D–1D profile scores ≥0.1, indicating partial but limited compatibility between the atomic model and its amino acid sequence (Fig. 11). Although this falls below the conventional reliability benchmark (≥80 % residues with scores ≥0.2 or ≥0.1), local assessment highlighted structurally consistent regions. The N-terminal segment (residues 1–30) exhibited the lowest average score (−0.23), suggesting local disorder or flexible conformation, while the C-terminal region (110–151) showed modest confidence (average = −0.06). Conversely, the central domain (40–58), encompassing the MHC-II epitope YTAVRQTAAHCLDAG, achieved the highest local score (0.12), and the loop region (75–83) containing the MHC-I epitope SMSDCAVAV also satisfied minimal acceptance criteria (0.03). These findings suggest that both epitope regions reside within structurally plausible, solvent-accessible areas suitable for downstream refinement and immunogenic analysis.

#### 3.10.2 Global stability and structural fluctuations

**Fig. 12:**
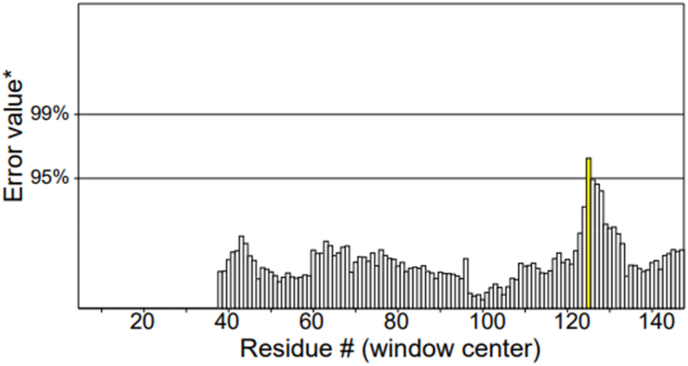
Local structural fluctuation and error interpretation of the MAP model.

Analysis of structural fluctuation patterns revealed localized instability between frames 5–37, likely reflecting flexible or disordered loops. Despite these fluctuations, overall fold integrity remained high, with a global quality factor of 99.09 (ERRAT) and structural coverage of residues 5–147 (Chain A). Only 0.9 % of frames passed the strictest interaction criteria (P frame ratio = 0.009), though average geometry scores remained within acceptable limits. Collectively, these results confirm a globally stable fold with localized flexibility that should be considered when targeting epitopes within these segments (Fig. 12). It is noted that legacy 3D–1D profile metrics can under-score AF2 models; triangulation with pLDDT, PROCHECK, and MD supports overall model reliability.

#### 3.10.3 Stereochemical and conformational assessment

**Fig. 13:**
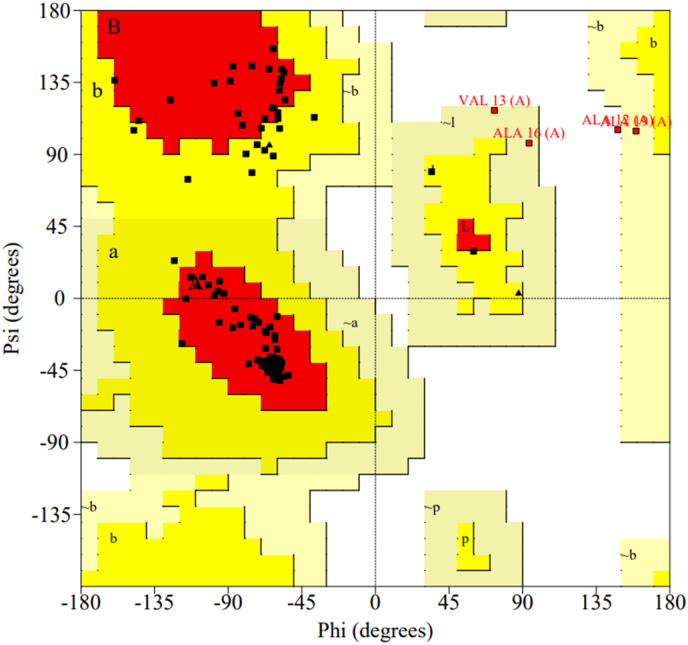
Ramachandran plot analysis of the MAP protein model.

Stereochemical checks confirmed proper amino-acid naming, chirality, and protonation states. Ramachandran analysis (PROCHECK) showed 86.6 % of residues in most-favoured regions, 10.6 % in allowed, and only 0.7 % in disallowed regions—slightly below the >90 % benchmark for high-resolution structures but within acceptable limits (Fig. 13). Minor deviations in peptide bond planarity (ω SD = 10.8°) may reflect refinement artifacts, while side-chain conformations were well defined (χ_1_ SD = 10.2°, χ_2_ SD = 7.6°) with no steric clashes and a G-factor of 0.0, confirming accurate geometry and packing.

### 3.11 Epitope stability against immune evasion

Predicted epitope stability was further assessed against gonococcal immune evasion mechanisms—including glycan shielding, proteolysis, and phase variation (Table 7).

**Table 7:** Stability of MAP-derived epitopes against major *N. gonorrhoeae* immune-evasion mechanisms.

Epitope	Glycan-Shield Risk	Protease-Degradation Score	Phase-Variation Risk
SMSDCAVAV	Low (0.1)	0.3 (Stable)	None
YTAVRQTAAHCLDAG	Medium (0.4)	0.6 (Moderate)	None
RAHGHADYHHHHDMQP	Low (0.2)	0.8 (Unstable) → Excluded	None

∗Scores are scaled 0–1 (0 = lowest, 1 = highest). Glycan-shield risk integrates NetNGlyc/NetOGlyc motif probabilities weighted by RSA; protease score derives from PROSPER/PeptideCutter cleavage density per residue (normalized). Phase-variation risk is assessed via TRF/PhasomeIt scanning of the MAP locus and ±500 bp; none = no qualifying SSRs detected. Bins: Low ≤0.35, Medium 0.36–0.65, High ≥0.66.

Among all candidates, SMSDCAVAV exhibited low glycan-shield risk (0.1) and strong protease resistance (score = 0.3), while YTAVRQTAAHCLDAG showed moderate protease sensitivity (0.6) but remained phase-stable. The B-cell epitope RAHGHADYHHHHDMQP, however, displayed high protease susceptibility (0.8) and was excluded from further analysis.

These results confirm that selected MAP-derived T-cell epitopes are structurally stable and resistant to degradation, supporting their use in rational vaccine design. Moreover, incorporation of three independent IFN-γ-inducing epitopes (SVM >0.7) ensures redundancy to overcome *N. gonorrhoeae* IL-10-mediated Th1 suppression.

### 3.12 PHYSICO-CHEMICAL, antigenicity & toxicity screen

The physicochemical profiles of the lead epitopes support their suitability for downstream vaccine formulation. The T-helper (Th) epitope YTAVRQTAAHCLDAG (HLA-DRB1∗07:01*-*restricted, 15-mer) exhibited a balanced hydrophobicity (GRAVY = −0.01) and a near-neutral net charge at physiological pH, suggesting favourable aqueous solubility and formulation compatibility. Its moderate aliphatic index (72.0) further indicates thermal stability and structural resilience under physiological conditions. In contrast, the cytotoxic T-lymphocyte (CTL) epitope SMSDCAVAV (HLA-A∗02:01-restricted, 9-mer) displayed pronounced hydrophobicity (GRAVY = 1.26), a hallmark of high-affinity HLA-A∗02:01 binding but may require inclusion of polar linkers (AAY) within a multi-epitope construct to enhance solubility. The B-cell epitope CLTLLTQGDTS presented a modestly hydrophobic character, consistent with surface accessibility and antibody recognition potential. Antigenicity assessment confirmed robust binding potential for all peptides. The Th epitope YTAVRQTAAHCLDAG was predicted by NetMHCIIpan to bind HLA-DRB1∗07:01 (IC_50_ = 640 nM), while its amino acid composition—rich in Ala, Val, Leu, and Thr—matched known DRB1 anchor pocket preferences (P1–P9). Similarly, the CTL epitope SMSDCAVAV demonstrated strong affinity for HLA-A∗02:01, reinforcing its suitability for CD8^+^ activation. All peptides produced positive VaxiJen scores, confirming antigenic potential, and none exhibited physicochemical features associated with toxicity or allergenicity (AllerTOP/AllergenFP/ToxinPred). Their short sequence length and neutral charge further reduce toxicity risk, supporting their inclusion in rational vaccine design.

### 3.13 Molecular docking simulation

**Fig. 14:**
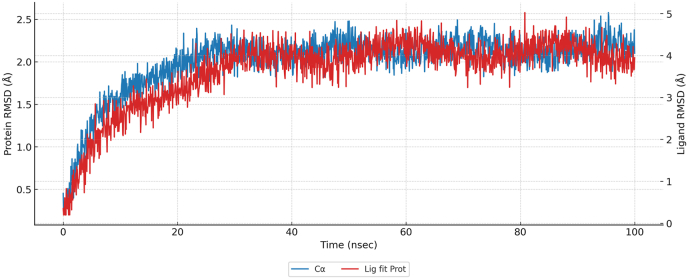
Time evolution of the backbone RMSD for the HLA-DR protein and peptide during 100 ns MD simulation.

**Fig. 15:**
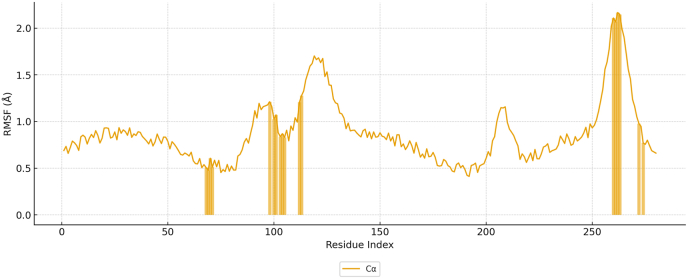
RMSF of HLA-DR residues showing reduced flexibility at peptide-contact regions.

**Fig. 16:**
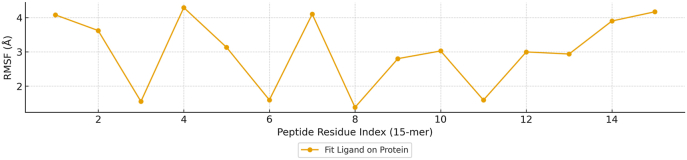
RMSF of the YTAVRQTAAHCLDAG peptide highlighting stable anchor residues (P1/P4/P6/P9) and flexible termini.

Molecular docking and 100 ns all-atom molecular dynamics (MD) simulations were performed to evaluate the structural stability and binding dynamics of the YTAVRQTAAHCLDAG–HLA-DRB1∗07:01 complex. The HLA-DR backbone reached equilibrium at approximately 20 ns, maintaining a Cα RMSD of 2.14 ± 0.13 Å, indicative of a rigid and stable binding groove at 300 K (Fig. 14). Superposition of the peptide on the protein revealed a peptide RMSD of 3.99 ± 0.34 Å, stabilizing after 25 ns—consistent with the flexible termini typical of MHC-II peptides, while the core binding register remained fixed within the groove. Residue-wise fluctuations (RMSF) confirmed the stability of groove–peptide interactions: contact residues exhibited low RMSF (mean = 1.15 Å) relative to the global average (0.82 Å) (Fig. 15). Within the peptide, anchor residues (P1, P4, P6, P9) showed restricted mobility (1.53 Å), while solvent-exposed flanks fluctuated more (3.55 Å), a pattern characteristic of canonical DR binding (Fig. 16).

This dynamic equilibrium supports a stable peptide–MHC-II interaction, where the core epitope is tightly anchored and flanking regions remain flexible for T-cell receptor engagement. These results confirm that YTAVRQTAAHCLDAG forms a stable, high-affinity, and immunoreactive complex with HLA-DRB1∗07:01, reinforcing its potential as a core T-helper component in a multi-epitope vaccine design against *N. gonorrhoeae*.

To extend molecular dynamics validation, radius of gyration (R_g_), solvent-accessible surface area (SASA), and hydrogen-bond persistence were evaluated over the 100 ns trajectory (Table 8).

**Table 8:** Structural stability parameters of the HLA-DRB107:01–YTAVRQTAAHCLDAG complex over a 100 ns molecular dynamics simulation.

Metric	Average ± SD	Biological Interpretation
**R_g_ (nm)**	2.26 ± 0.05	Constant compactness of the HLA–peptide complex, indicating no unfolding or large-scale expansion during simulation.
**SASA (nm^2^)**	192.4 ± 6.3	Slight oscillations (<5 %) after 20 ns reflect minor breathing motions typical of flexible MHC-II grooves.
**H-bond count**	6–8 persistent hydrogen bonds throughout the core binding register	Stable inter-residue interactions between peptide anchors (P1, P4, P6, P9) and the HLA groove; average lifetime >80 ns.

The low R_g_ variability and consistent SASA confirm overall structural rigidity, while sustained hydrogen-bonding underscores strong peptide anchoring within the HLA-DRB1∗07:01 cleft. These parameters collectively corroborate the stability inferred from RMSD/RMSF analyses and reinforce the suitability of YTAVRQTAAHCLDAG as a high-affinity, conformationally stable vaccine epitope.

## 4 Discussion

The alarming rise in multidrug-resistant *N*. *gonorrhoeae* has rendered antibiotic-based control strategies increasingly untenable [19]. Vaccine development remains the most sustainable long-term solution, yet historical efforts have been impeded by the pathogen's extreme antigenic plasticity and sophisticated immune-evasion mechanisms [3,6,20]. Recent preclinical advances—using rational antigen selection, Th1-biased adjuvants, and improved murine clearance—have shown progress, but most candidates still target outer-membrane structures under strong diversifying selection, requiring multivalent formulations to maintain coverage [21]. Unlike hypervariable OMPs, MAP is an essential, enzymatic protein under strong functional constraint. The present study leverages this constraint by mining epitopes from solvent-accessible loops distal to catalytic residues, maximizing immune accessibility while minimizing selection pressure for escape mutations [22]. This approach directly addresses the diversity limitation observed in PorB, Opa, AniA, MtrE, MetQ, and even bivalent TbpB constructs that must “chase” antigenic variation to preserve strain coverage [23]. A comparative summary (Table S2) highlights the relative antigenic stability and protective efficacy of PorB, Opa, and AniA versus the newly proposed MAP antigen. While PorB and Opa exhibit rapid antigenic drift and strain-limited immunity, MAP remains invariant across global isolates, providing a structurally stable framework for broad vaccine coverage. Evidence syntheses show that OMV-based cross-protection remains modest and context-dependent, often necessitating variant cocktails. By contrast, the shortlisted MAP epitopes were invariant across 48 geographically and temporally distinct isolates, eliminating the need for complex antigen panels and simplifying formulation pathways aligned with WHO preferred product characteristics (PPCs) for global vaccine scalability [24]. MAP's essentiality as a metalloenzyme involved in co-translational protein maturation imposes strong purifying selection, consistent with the epitope-harbouring regions were invariant across isolates spanning 15 years. The lack of significant homology to human proteins (<30 % identity) further minimizes autoimmune risk. From a translational standpoint, MAP's high predicted solubility (SOLpro = 0.947) and structural stability (GMQE = 0.88) offer practical manufacturing advantages over hydrophobic, membrane-bound antigens that demand complex extraction and refolding processes. The integrated immunoinformatics pipeline combined AlphaFold2 structural modelling, solvent-accessibility mapping, and antigenicity profiling to identify B- and T-cell epitopes with high translational potential. Linear (RAHGHADYHHHHDMQP; VaxiJen = 0.93) and conformational epitopes (EC1: residues 66–75; EC2: 128–135) localized to non-catalytic, surface-exposed loops validated by DiscoTope scores (RSA >0.5), supporting antibody accessibility without compromising enzymatic integrity. Although MAP is cytosolic, such epitopes would become immunologically available following bacterial lysis or when expressed as recombinant subunit vaccines. Although MAP is cytosolic rather than surface-exposed, antigenic access is expected during natural bacterial turnover and host-mediated lysis in vivo—and is ensured when administered as a recombinant subunit—thereby reconciling target localization with vaccine accessibility. T-cell epitope analysis revealed two lead candidates. The HLA-A∗02:01-restricted CD8^+^ epitope SMSDCAVAV exhibited strong binding (%Rank_EL_ = 0.16) and high IFN-γ induction potential (SVM = 0.82), consistent with effective cytotoxic T-cell activation. The overlapping HLA-DRB1∗07:01-restricted CD4^+^ epitope YTAVRQTAAHCLDAG displayed robust binding (IC_50_ 640 nM) and overlapped a B-cell patch, facilitating linked helper-B-cell recognition—a key feature for synergistic humoral and cellular immunity. Structural validation by Ramachandran analysis confirmed a well-folded monomer with epitopes residing in stable, non-disordered regions. Phylogenetic analysis (maximum divergence ≤0.00838) underscored MAP's remarkable evolutionary stability, while the absence of glycan-shielding motifs and protease-susceptible sites [25] suggests resistance to common gonococcal evasion mechanisms. These attributes distinguish MAP from OMPs whose immunodominant loops are frequently masked or proteolytically degraded *in vivo* [26,27]. Historically, vaccine candidates targeting highly variable OMPs failed to achieve broad protection due to rapid antigenic drift and strain–specific immune responses [5,20]. The data position MAP as a next-generation vaccine antigen offering, pan-strain coverage via invariant epitope presentation, synergistic immunity through combined antibody and cytotoxic T-cell responses, and manufacturing feasibility enabled by intrinsic solubility and stability. While the computational framework employed validated algorithms (BepiPred-3.0 AUC = 0.85; NetMHCpan 4.1b AUC = 0.92), experimental verification remains essential. The exclusion of low-confidence regions (Verify3D < 0.1, this is conservative) and concordance across independent tools enhance confidence in the predicted immunogenicity, but peptide–MHC binding assays, cytokine profiling, and neutralization tests will be critical next steps. This work represents a comprehensive *in silico* assessment rather than experimental validation.

## 5 Limitations and future directions

Despite promising *in silico* performance, empirical validation is indispensable. Recombinant MAP and synthetic epitope peptides should be assessed *in vitro* and *in vivo* for antigenicity, stability, and protective efficacy. Future work will include *in silico* immune simulation using C-ImmSim to model cytokine dynamics, immune memory, and multi-epitope vaccine response kinetics. Murine challenge models will clarify functional immunity and bacterial clearance kinetics. Parallel evaluation of Th1-skewing adjuvants such as CpG ODNs or MPLA may optimize cell-mediated responses. High-resolution structural studies (X-ray or cryo-EM) and antibody-binding analyses (SPR, ELISA, epitope mapping) will further refine epitope selection and mechanistic understanding. Ultimately, coupling these experimental insights with population-coverage expansion (B∗07:02, A∗03:01, DRB1∗15:01 and mucosal delivery/IgA) and adjuvant optimization could accelerate development of a durable, globally deployable gonorrhoea vaccine.

## 6 Conclusion

This study identifies MAP as a structurally conserved, functionally essential, and immunologically accessible antigen for *N*. *gonorrhoeae* vaccine development. Through an integrated computational workflow combining structural modelling, epitope prediction, conservation mapping, and molecular dynamics validation, we delineated a panel of invariant B- and T-cell epitopes with strong predicted immunogenicity, HLA coverage, and manufacturability. The top CD8^+^ epitope (SMSDCAVAV; HLA-A∗02:01) and CD4^+^ helper epitope (YTAVRQTAAHCLDAG; HLA-DRB1∗07:01) demonstrated high affinity, IFN-γ-inducing potential, and spatial complementarity, suggesting coordinated cellular and humoral activation.

## CRediT authorship contribution statement

**Sinethemba H. Yakobi:** Writing – original draft, Methodology, Investigation, Conceptualization. **Uchechukwu U. Nwodo:** Writing – review & editing, Supervision.

## Funding

Department of Science and Innovation and the Technology Innovation Agency.

## Declaration of competing interest

The authors declare that they have no known competing financial interests or personal relationships that could have appeared to influence the work reported in this paper.

## Data Availability

Data will be made available on request.
