# Supplementary material for: Structure-guided in silico design of a methionine aminopeptidase–derived multi-epitope vaccine candidate against Neisseria gonorrhoeae
Source: Biochem Biophys Rep. 2025 Oct 23;44:102323. doi: 10.1016/j.bbrep.2025.102323 (PMC12593588; doi:10.1016/j.bbrep.2025.102323)
Supplement: Multimedia component 1 [file mmc1.docx]

# Supplementary Material

**Table S1.** Phylogenetic divergence of *Neisseria gonorrhoeae* MAP sequences relative to reference (Q5F5E6).

| **Sequence ID** | **Accession (Contig)** | **Start–End (aa)** | **Divergence from reference** | **Clade Assignment** | **Interpretation** |
| --- | --- | --- | --- | --- | --- |
| Q5F5E6_NEIG1 | UniProt | Full length | 0.00838 | Closest | Reference sequence |
| NZ_BLUX01000069.1 | 121–900 | 121–900 | 0.00838 | Closest | Most similar sequence to reference |
| NZ_BLUR01000078.1 | 1–553 | 1–553 | 0.00510 | Slightly Divergent | High similarity with minor variation |
| NZ_JBGMMB010000071.1 | c8046–7492 | c8046–7492 | 0.00201 | Slightly Divergent | Minor divergence from reference |
| NZ_CP045832.1 | 1817823–1818602 | 1817823–1818602 | 0.00201 | Slightly Divergent | Highly conserved, near‑reference |
| NZ_JBGMCW010000072.1 | c8156–7580 | c8156–7580 | 0.00079 | Most Divergent | Edge of clade, small divergence |
| NZ_CAXETE010000074.1 | c8054–7476 | c8054–7476 | 0.00042 | Most Divergent | Potential variant or sequencing artifact |
| NZ_BLUN010000072.1 | 1–593 | 1–593 | 0.00023 | Slightly Divergent | Conserved |
| NZ_JADPX010000062.1 | 1–599 | 1–599 | 0.00000 | Identical | Perfectly conserved |
| NZ_BLUOA01000075.1 | c9168–8574 | c9168–8574 | 0.00000 | Identical | Identical to reference |
| NZ_BLVM01000096.1 | 1–599 | 1–599 | 0.00000 | Identical | Identical |
| NZ_BLUF01000841.1 | c9225–8628 | c9225–8628 | 0.00000 | Identical | Identical |
| NZ_BLUO01000081.1 | c8132–7535 | c8132–7535 | 0.00000 | Identical | Identical |
| NZ_BLUV01000094.1 | 1–597 | 1–597 | 0.00000 | Identical | Identical |
| NZ_BLTU01000743.1 | c9221–8627 | c9221–8627 | 0.00000 | Identical | Identical |
| NZ_BLUW01000084.1 | 1–599 | 1–599 | 0.00000 | Identical | Identical |
| NZ_BLTK01000078.1 | c9092–8514 | c9092–8514 | 0.00000 | Identical | Identical |
| NZ_BLUY01000008.1 | c9097–8514 | c9097–8514 | 0.00000 | Identical | Identical |
| NZ_JBGMAU010000081.1 | c8248–7682 | c8248–7682 | 0.00000 | Identical | Identical |
| NZ_BLUQ01000036.1 | c9071–8514 | c9071–8514 | 0.00000 | Identical | Identical |
| NZ_BLUO01000084.1 | 1–593 | 1–593 | 0.00000 | Identical | Identical |
| NZ_BLVG01000224.1 | c650–110 | c650–110 | 0.00000 | Identical | Identical |
| NZ_BLUY01000844.1 | 1–593 | 1–593 | 0.00000 | Identical | Identical |

**Table S2.** Comparative summary of antigenic stability and protective efficacy of selected *N. gonorrhoeae* vaccine antigens.

| **Antigen** | **Localization / Function** | **Antigenic Variability** | **Reported Protective Efficacy in Animal Models** | **Structural / Immunological Limitations** | **Comparative Advantage of MAP** |
| --- | --- | --- | --- | --- | --- |
| **PorB** | Major outer-membrane porin; facilitates nutrient transport | High — extensive allelic diversity (>15 serotypes) and immune-driven recombination | Partial, strain-specific protection (<40% bacterial clearance) | Phase variation, glycan masking, complement inhibition; low cross-strain immunity | MAP is enzymatically essential and invariant across isolates; not subject to phase variation or surface masking |
| **Opa** | Opacity-associated outer-membrane adhesin mediating epithelial invasion | Very high — subject to phase variation via pentameric repeats | No consistent protection; antibodies often non-neutralizing | Rapid on/off switching, host mimicry, immune suppression | MAP has stable expression and exposes conserved loops suitable for durable immune targeting |
| **AniA** | Nitrite reductase supporting anaerobic respiration and biofilm formation | Moderate — several polymorphic variants (85% identity across strains) | Partial reduction in colonization (50% in murine models | Surface glycosylation and oxygen-regulated expression reduce antigen availability | MAP is cytosolic but accessible upon lysis; structural rigidity enhances epitope preservation |
| **MAP (This study)** | Cytosolic methionine aminopeptidase; co-translational N-terminal processing enzyme | Very low — invariant across 48 global isolates (divergence ≤ 0.008) | *In silico* data predict broad HLA coverage; experimental validation pending | No *in vivo* data yet; accessibility depends on bacterial lysis or recombinant delivery | Structurally conserved, soluble, and manufacturable; elicits dual CD4⁺/CD8⁺ epitope responses |

******* *Protective efficacy” reflects percent bacterial clearance or reduction in colonization relative to control.*
